# Visual Processing by Hierarchical and Dynamic Multiplexing

**DOI:** 10.1523/ENEURO.0282-24.2024

**Published:** 2024-11-12

**Authors:** Mathilde Bonnefond, Ole Jensen, Tommy Clausner

**Affiliations:** ^1^Lyon Neuroscience Research Center, Computation, Cognition and Neurophysiology (Cophy) team, INSERM UMRS 1028, CNRS UMR 5292, Université Claude Bernard Lyon 1, Bron Cedex 69675, France; ^2^Centre for Human Brain Health, School of Psychology, University of Birmingham, Birmingham B15 2TT, United Kingdom

**Keywords:** alpha, attention sampling, hierarchical multiplexing, phase coding, saccades, theta

## Abstract

The complexity of natural environments requires highly flexible mechanisms for adaptive processing of single and multiple stimuli. Neuronal oscillations could be an ideal candidate for implementing such flexibility in neural systems. Here, we present a framework for structuring attention-guided processing of complex visual scenes in humans, based on multiplexing and phase coding schemes. Importantly, we suggest that the dynamic fluctuations of excitability vary rapidly in terms of magnitude, frequency and wave-form over time, i.e., they are not necessarily sinusoidal or sustained oscillations. Different elements of single objects would be processed within a single cycle (burst) of alpha activity (7–14 Hz), allowing for the formation of coherent object representations while separating multiple objects across multiple cycles. Each element of an object would be processed separately in time—expressed as different gamma band bursts (>30 Hz)—along the alpha phase. Since the processing capacity per alpha cycle is limited, an inverse relationship between object resolution and size of attentional spotlight ensures independence of the proposed mechanism from absolute object complexity. Frequency and wave-shape of those fluctuations would depend on the nature of the object that is processed and on cognitive demands. Multiple objects would further be organized along the phase of slower fluctuations (e.g., theta), potentially driven by saccades. Complex scene processing, involving covert attention and eye movements, would therefore be associated with multiple frequency changes in the alpha and lower frequency range. This framework embraces the idea of a hierarchical organization of visual processing, independent of environmental temporal dynamics.

## Significance Statement

The complexity of natural environments necessitates highly adaptable mechanisms for processing single and multiple stimuli. Neuronal rhythmical fluctuations present an ideal solution for such flexibility in neural systems. We propose a framework for attention-guided processing of complex visual scenes in humans, utilizing rapid fluctuations of activity at multiplex frequencies and phase coding schemes. Different elements of single objects would be processed within a single alpha activity cycle (7–14 Hz), forming coherent representations, while multiple objects would be processed across multiple cycles. An inverse relationship between object resolution and attentional spotlight size ensures independence from object complexity. This framework provides a novel computational perspective on visual processing and is highly capable of reconciling previous findings and providing highly informative testable hypotheses.

## Functional roles of oscillatory dynamics at multiple frequencies in visual scene processing

Visual scene processing is one of the fundamental functions of the brain, and yet its in depth algorithmic implementation in the brain remains to be fully understood. Here we propose a novel framework for the implementation of visual scene processing where the mechanisms are anchored in highly flexible cortical dynamics. Repetitive cortical dynamics are traditionally referred to as neuronal “oscillations.” Even though the term implies a sustained rhythmic activity, we would like to emphasize that in the present context, neuronal oscillations are understood as nonsustained—probably burst-like—flexible adjustments of cortical excitability. Hence, we would rather use the term “cortical fluctuation” in the e.g., alpha band or e.g., alpha fluctuations. We will however adopt the common frequency naming scheme (delta, theta, etc) to facilitate reading and refer to “cortical oscillations” when referencing the respective literature.

**Figure 1.**
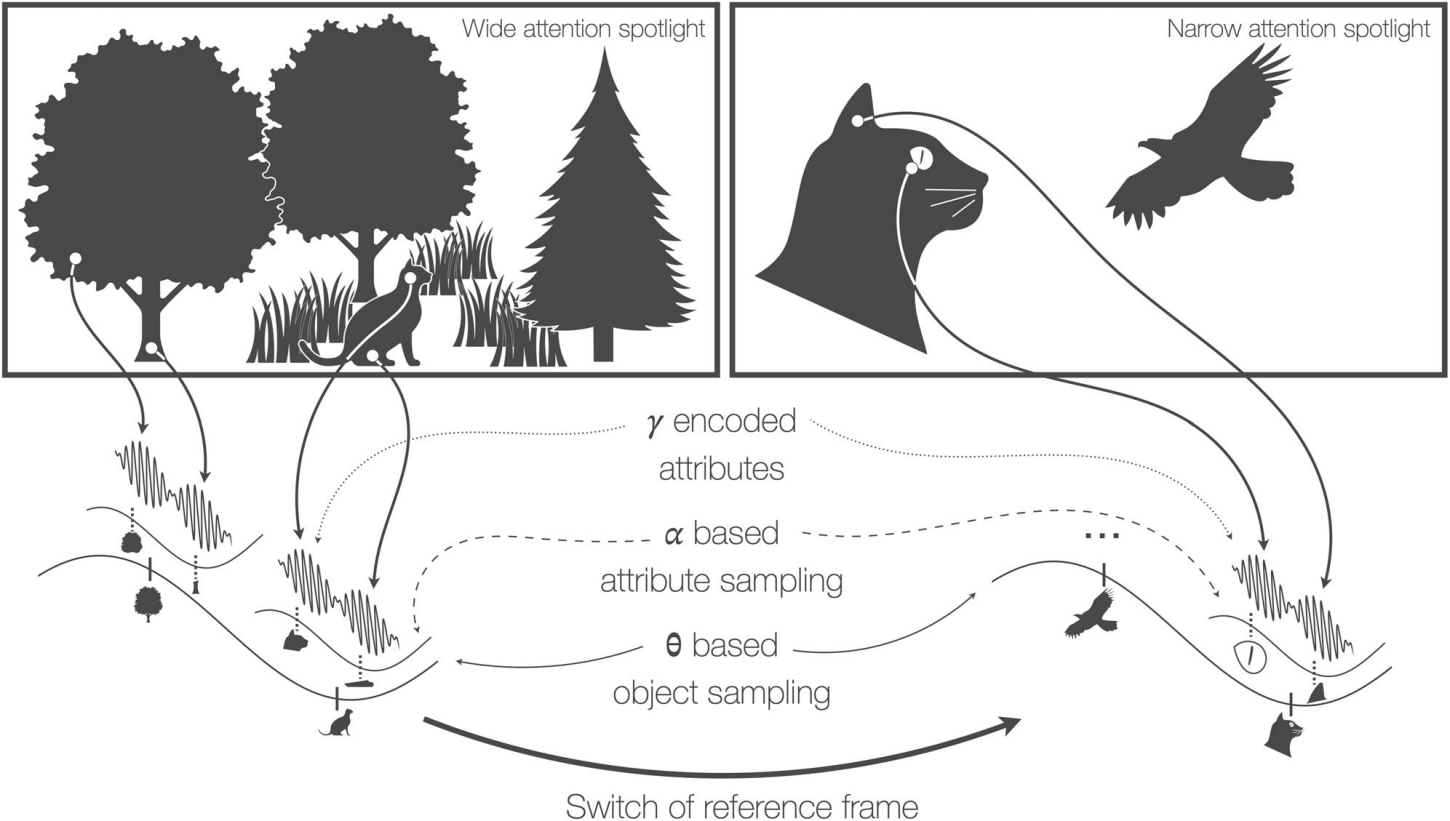
Flexible oscillatory multiplexing and phase coding allows for processing single and multiple objects in different frames. ***left***: wide attentional spotlight. A natural scene is composed of one or multiple objects. Here, you are watching your cat playing in the garden between trees. Both the trees and the cat are composed of several attributes like stem and crown (trees) or head and paws (cat). We suggest that each of those attributes is reflected on the neuronal level by distinct bursts of gamma band activity. Each burst thereby is tightly linked to a distinct phase of alpha band activity, whereby the respective features are “ranked” along alpha’s phase gradient, where attributes exerting higher neuronal activity (e.g., due to higher levels of saliency, attention, etc.) are processed earlier in the alpha phase, because they are able to overcome the pulsed inhibition earlier in the cycle. This mechanism would be at play for grouping (within an alpha cycle) the different elements of an object, while taking into account the specificity of each attribute. Similarly, each separate object in turn is coded along the theta/delta phase gradient (again depending on the level of excitation). Alternatively, this change of excitability could also be caused by saccadic eye movement itself. ***right***: narrow attentional spotlight. If the focus of attention is more narrow (i.e., “zoomed in” attentional spotlight), we suggest that the frame of reference shifts, such that former attributes can become objects that are in turn composed of smaller attributes, allowing for a higher level of detail. Here, the focus of attention shifted towards the head of the cat, which is threatened by a predatory bird. In general, the same principles of phase coding apply, but now the level of detail has increased (i.e., the cat’s eyes become attributes and the cat’s head the object). Note that dynamic fluctuations are represented as sinusoidal oscillations only for illustrative purposes.

According to Henderson and Hollingworth (1999), a visual scene can be considered as a semantically coherent view of some real-world environment. This view is composed of a background and one or more distinct spatially arranged objects. Furthermore, Epstein (2005) derives from this definition that objects can be described as “spatially compact entities [within scenes]”. We extend the notion and further argue that an object is characterized by one or multiple attributes. For example, the scene of watching your cat playing in the garden would be composed of multiple objects, namely the trees, the cat, potentially uncut patches of your lawn, etc. In turn the cat “object” for example is composed of several attributes like its head, paws, ears, whiskers, eyes, etc. (see Fig. 1 left for illustration). Note that we would like to make a distinction between attributes that form an object (but are often called features in the literature) and true features as inherent to attributes. Color or shape would hence be a feature of an attribute, which in turn would be referred to as “feature” of an object in parts of the literature (e.g., the cat’s eye is an attribute of the cat, but the eye being e.g., green or almond shaped would be a feature).

We suggest that in order to effectively process visual scenes, the brain needs to translate the spatial structure of the visual input into a spatio-temporal code that is independent of the temporal dynamics of the environment (see Schroeder and Lakatos, 2009 for a hierarchical dynamic model of neuronal processing considering the temporal structure of the environment).

First, to differentiate between multiple objects, the brain must integrate each object’s specific attributes as belonging to that object. For example, if the cat and a tree overlap in the scene, the brain must recognize the cat’s attributes as separate from the tree’s attributes. This integration is a higher-level process than feature binding (e.g., color and shape) of a single object in a simple environment (Di Lollo, 2012). Feature binding instead is proposed to be processed at an early stage possibly via transient activities (Wutz and Melcher, 2014; Lowet et al., 2018; Resulaj et al., 2018), by neurons specifically coding for conjunctions of features at the same location or specific networks of neurons after learning (Seymour K et al., 2010).

We propose that each attribute must be represented as a distinct entity in order to allow the brain to assign it to the corresponding objects, because the processing of multiple attributes of different objects using partially the same neuronal substrate due to overlapping receptive fields and visual stream convergence (“bottleneck issue” Rousselet et al., 2004) at the same moment in time would render attribute differentiation (and hence assignment) rather challenging. This of course requires prior knowledge of objects and attributes that typically belong to certain objects. The same is true for processing multiple objects. One solution to this bottleneck issue is that information processing is handled serially by the brain to avoid mixed attribute assignment when relying on the same neuronal substrate (when processing multiple attributes or objects). From this serial structure, we can derive that information is unlikely processed time-independent (as it must occur one after another).

Finally, given the dynamic nature of natural scenes (e.g., a predatory bird could approach the cat; see Fig. 1 right), dynamic changes of internal goals (e.g., switching towards the cat’s eyes to determine whether it has detected the bird) and the described need for a serial organization of information, the brain is required to rapidly adapt its temporal processing to the new goals. While the change of the scene itself is determined by external processes, a switch of internal goals might as well require rearranging the reference frame. Here, the frame of reference refers to the entirety of the visual input the observer is actively processing. However, we assume an inverse relationship between spatial coverage and visual resolution, such that the more complex or wider the visual scene, the less detailed each object and attribute can be processed and vice versa (e.g., when “zooming in” to a part of the scene it can be processed in more detail but at less spatial coverage). In the present example, this could mean shifting the focus of attention (and thus the frame of reference) from the cat to the cat’s eyes in order to accommodate the new internal state (determining whether the cat has seen the bird). In general, attention has a large impact on the processed information by either covertly identifying an object of interest (e.g., the predatory bird which poses a threat to the cat) or overtly shifting the eye’s positions towards a new target. Thus, visual processes are inevitably tightly linked to the eye’s position or eye movements (saccades) and hence temporal dynamics of the environment impact saccadic behavior. The inverse is also true, where e.g., a change of internal goals might cause saccades to change the exact visual input (Parr and Friston, 2019). In both cases (scene change or internal goals change), the serial processing of the brain must adapt accordingly (and hence cannot be static). A *dynamic* temporal processing scheme would inevitably be required.

Here, we argue that this organization scheme is realized by a temporal organization based on hierarchical and dynamic fluctuations of neuronal excitability. More specifically, we propose that **multiplexing**, where numerous information streams share a common neural substrate and organized by “rhythmical” activity at multiple frequencies, might be crucial for actively representing and perceiving information from complex visual scenes.

The proposed hierarchical framework (see Fig. 1) comprises multiple aspects, which we will present in the following. Direct and indirect evidence will be presented thereafter to support those ideas and the respective conclusions drawn from it. Lastly, we will provide a set of testable predictions that would consequently follow. In general, we will treat high and low frequency oscillations as functionally distinct, where the first (beta and gamma band activity) will be more related to stimulus-related processes themselves and the latter (delta, theta, alpha fluctuations) more to the organization of those processes.

In our framework, a distributed firing code organized by gamma band synchronization (>30 Hz) would be related to the processing of attributes of a single object such that each gamma burst reflects information about one given attribute of one given object at a given point in time. As such, they could keep representations of attributes apart, as proposed by Lisman and colleagues (e.g., Lisman and Jensen, 2013). Those gamma bursts would be nested within alpha band activity (7–14 Hz). However, the exact details of how gamma band activity is involved in visual scene processing is not the focus of this publication and the exact nature of gamma band activity does not change the here proposed model (see also below).

Alpha band oscillations have been associated with pulses of inhibition (Klimesch et al., 2007; Jensen and Mazaheri, 2010; Mathewson et al., 2011). This refers to the idea that rhythmic inhibition times the flow of information such that if inhibition is high, no information can be transferred to other neurons and windows of lower inhibition determine at which points in time information can be transferred (see e.g.,Fig. 1 in Klimesch et al., 2007). We propose that the variation of strength of inhibition within a single alpha cycle (or pulse) forms a gradient that in combination with the strength of the incoming signal (e.g., determined by saliency, relevance or distance from eyes focus, etc.) dictates the temporal order at which this information is processed (Jensen et al., 2014). Strong signals are able to overcome the pulsed inhibition earlier in the cycle than less strong inputs. Within a single alpha cycle, the object’s attribute composition would hence be reflected by the temporally sorted gamma bursts. This means that the period of a single alpha cycle (determined by its frequency) or the window of opportunity at which the incoming signal actually can overcome the inhibition (determined by the power) have an impact on the amount of information that can be processed within a single cycle. In turn, this has an impact on the resolution of the processed input, since only so much information can fit a single cycle (again, depending on frequency and power).

Similar to the processing of groups of attributes within an alpha cycle, multiple objects would be encoded in delta or theta fluctuations (1–7 Hz) along their respective (inhibitory) gradients. Those might be associated with pulses of inhibition too and might support the organization of multiple objects exploiting different levels of attention or salience (most salient first). It has been shown recently that the theta rhythm was modulated by attention, i.e., it was lower in the visual hemifield processing the attended stimulus (Spyropoulos et al., 2018) and that had reduced power in FEF/LIP following cue onset (Fiebelkorn et al., 2018). If each object is reflected by the proposed alpha mechanism, then alpha fluctuations would be nested in delta/theta. Hence, delta or theta fluctuations would encode “object collections”. Changes in frequency and/or power would—similar to alpha fluctuations—impact the number of objects that can be processed within a single cycle (see Fig. 1 left bottom). Those processes are tightly linked to attention. An overt shift of attention would be accompanied by saccadic eye movement. The theta/delta band phase (between objects) or the alpha band phase (within objects) would be linked to the onset of the saccades. The main difference would lie within the state of the internal goal of the observer: For single objects, for instance, a very detailed processing of all the object’s attributes would lead to a stronger link between the alpha phase and the onset of saccades, whereas if mostly the shift of attention between objects drives the task (e.g., when detecting an outlier object among a set), the theta/delta phase would act as the main driver (see also Schroeder et al., 2010 for an overview on active sensing). Alternatively, saccades could be the mechanism that create the slow fluctuation of excitability instead of being dependent on it. The stimulus presented at the fovea after a saccade is processed first (Xiao et al., 2024) and then subsequent processing of parafoveal stimuli (Pan et al., 2021) might take place in the next alpha/beta cycle, before the onset of the next saccade. The dependence on theta/delta or alpha fluctuations would also apply to covert shifts of attention.

In a highly dynamic and complex environment, the processing of visual scenes must be able to rapidly adapt, and thus respective processes requires a high amount of flexibility. We state that it would be detrimental for efficient processing if oscillatory activity exposes too many regularities in frequency, amplitude, wave-form or phase. More precisely, we would like to explicate that these oscillations at different frequencies should adapt very rapidly, e.g., with a change in wave-form, amplitude and frequency, to match the specific computational needs that emerge in a complex dynamic environment, and therefore allow the system to be highly adaptive similarly to the phase coding observed in the hippocampus of bats (Eliav et al., 2018; Bush and Burgess, 2019). Note, that oscillations are not expected to be perfectly sinusoidal, but rather are expected to appear more like an inhibitory sawtooth wave comparable to the hippocampal theta rhythm as observed in rats (see, e.g., Cole SR and Voytek, 2017). In the present context, the sawtooth shape is thought to be the result of an initial pulse of inhibition that decays over time, providing the base for gradient based coding. In general, the here proposed framework can be seen as independent of requirements on stationarity or sinusoidal shape of the underlying waveforms, as the only two strict requirements are (a) a gradient (or multiple) of activation along which the respective information is processed and (b) a certain time span that is covered by this gradient. Non-sinusoidal waveforms might influence the exact timing at which a certain inhibitory value can be overcome and the amount of information that can be fit into the duration of the gradient.

For both, delta/theta-based object and alpha fluctuations based attribute encoding, the reference frame plays an important role. Specifically, what is considered an object depends on what is within the focus (of attention). Given the aforementioned example, the cat could be considered an object, but furthermore that cat’s head could be considered an object as well when focusing on this particular part. In the presented framework, this would mean that while in the first case the cat’s head would be considered an attribute, in the latter case the head would “turn into” an object with its attributes eyes, nose, etc. However, this would not change the principle mechanism as proposed, but rather would be a shift in reference frame (see Fig. 1).

The proposed mechanisms are complex, and it is challenging to determine how these rhythms are generated in which network. However, based on the literature, we suggest that interaction within the different visual regions along the hierarchy (Michalareas et al., 2016) are crucial, including higher order regions such as the parahippocampal place area (PPA) and fusiform face area (FFA) and low order areas such as V1, which receives feedback from higher order regions and in which decoding of complex stimulus can be performed (Ayzenshtat et al., 2012; Petro et al., 2013; Wang C et al., 2020). Given the role of prior knowledge and attention in the process, frontal regions as well as the hippocampus could be involved as well. We suggest that higher order regions such as PPA are involved in organizing the processing and as such in controlling the slow rhythm. In line with this idea, while slow rhythms are observed in all visual areas (Van-Kerkoerle et al., 2014; Bastos et al., 2015; Spyropoulos et al., 2018), it becomes prominent in higher order regions and the hippocampus (Mahjoory et al., 2020; Capilla et al., 2022). This might occur in anticipation or following a fast coarse processing of the scene. Alpha rhythm could be controlled in a more restricted network depending on the object processed (e.g., faces). In turn, gamma would be expected to be modulated more locally (Buzsáki and Wang, 2012; Spaak et al., 2012).

## Evidence and reinterpretation: phase-dependent visual object processing revisited

In this section, we will present evidence at the behavioral level and from neuronal recordings. Behavioral rhythms can only be seen as a proxy for the underlying neuronal activity, but have successfully been linked to electrophysiological data.

### Gamma oscillations at different frequencies are associated with attributes processing

Although the proposed model does not necessarily rely on the presence of gamma band fluctuations as distributed spiking along the alpha phase might yield comparable outcomes, gamma band activity has been related to the separation of stimulus information or the coordination of spiking activity (see e.g., Lisman and Jensen, 2013).

Gamma oscillations have traditionally been associated with the processing of stimulus features (Bertrand and Tallon-Baudry, 2000; Fries, 2005). However, it has been shown that different objects or different parts of an object could produce gamma oscillations at different frequencies, e.g., due to differences in contrast, orientation or eccentricity (Hadjipapas et al., 2015; Hermes et al., 2015a; Lowet et al., 2015). This finding has challenged ideas on the role of gamma oscillations in binding different parts (see also Roelfsema et al., 2004; Jia X et al., 2013; Ray and Maunsell, 2015; Roelfsema, 2023) or brain communication (Hermes et al., 2015b). In addition, it could be shown that saliency or relevance impacts on the gamma frequency as well (e.g., Bosman et al., 2012). However, here, a difference in frequency would not compromise the proposed mechanism of gamma bursts being encoded along the alpha phase. In contrast, it could even aid the proposed framework, since a difference in frequency for different encoded attributes would make a distinction of those attributes easier or reflect respective processes. Combined with the idea of increased gamma frequency by saliency (and hence a more excited neuronal substrate), higher frequency gamma bursts would be encoded earlier in the alpha phase.

### Alpha: one cycle, one object

In a nutshell, the here presented framework predicts that one coherent object would be processed within one single alpha cycle.

Direct evidence for this idea would mean mapping attributes of one object to one alpha cycle, whereas attributes belonging to different objects would be mapped to different alpha cycles. To our knowledge, this kind of evidence is mostly lacking in the literature.

**Figure 2.**
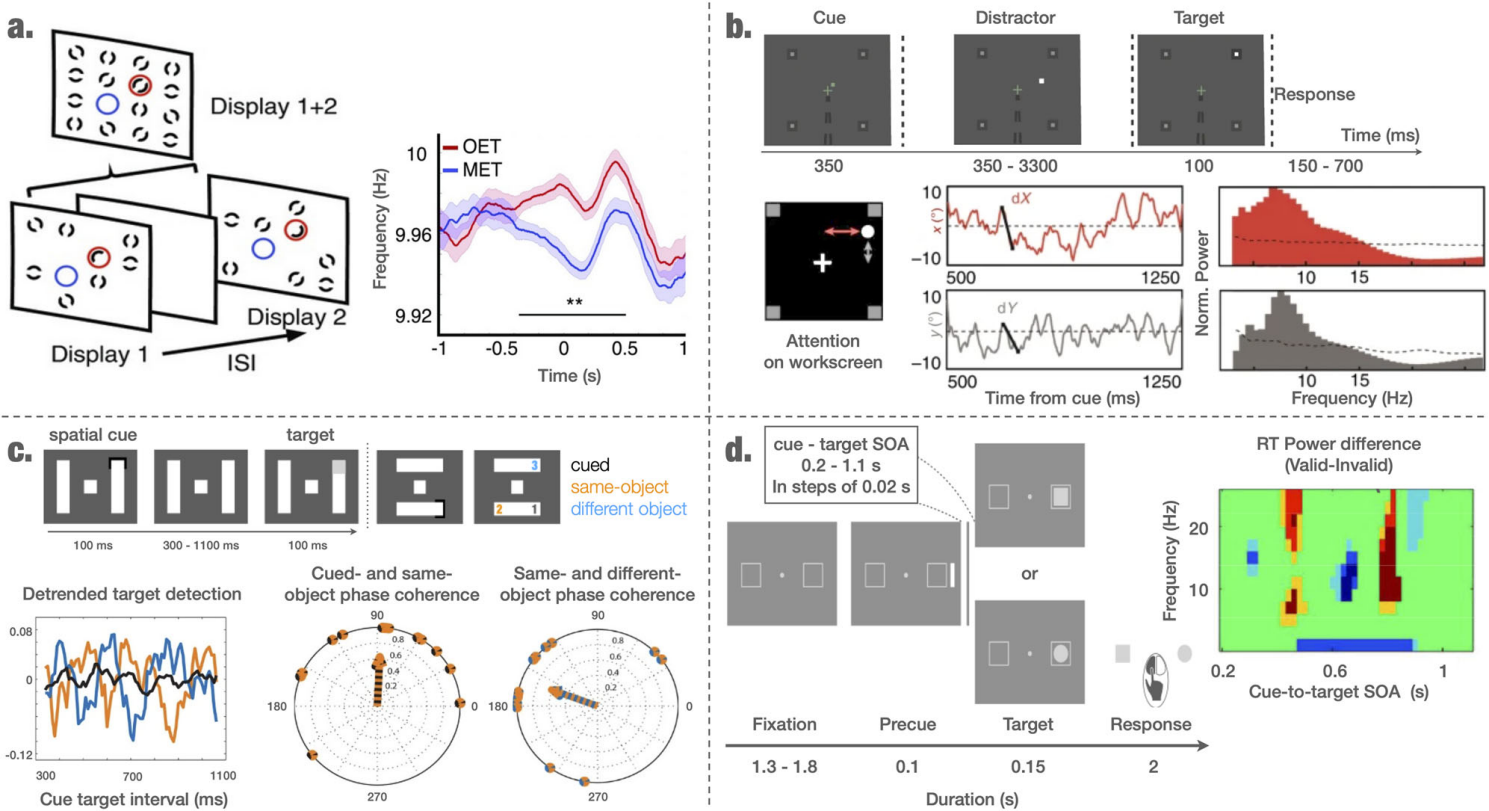
Evidence supporting the framework ***a***. The exact frequency of cortical oscillations in anticipation of the stimulus depends on task instructions (from Wutz et al., 2018). Participants were presented with two consecutive frames, separated by an inter stimulus interval (ISI). Each frame would display multiple graphical elements, and subjects were asked to either report an element absent in both frames (missing-element task or MET) or an element that was displayed in one half during the first frame and the other half during the second frame (odd-element task or OET). While the MET required cross-frame *integration*, the OET required cross-frame *segregation*. The authors showed that during the MET, the observed cortical frequency response (in the alpha range) was significantly lower in frequency as compared to the OET. This implies that the frequency of slow oscillations could be top-down controlled in order to favor integration of stimuli (with slower frequency) or segregation of the stimuli (with higher frequency). ***b***. The attentional spotlight samples spatial locations at alpha frequency (from Gaillard et al., 2020). In a 100% validly cued task, monkeys had to detect a target randomly presented at the cued location following the cue (with distractors presented in some trials). The spatial position of the attentional spotlight, decoded from the multi-unit activity in both the *x* and *y* directions, exhibited an 8–12 Hz rhythm. ***c***. Target detection times follow an 8 Hz behavioral rhythm within the same object and a 4 Hz rhythm between objects, indicating different sampling frequencies for within and between object sampling (from Fiebelkorn et al., 2013). After central fixation, a cue (75% validity) indicated to the participants that a target could appear at the cued location. In the remaining 25% of cases, the target could instead appear at a different location within the same object or within a different object. The distance to the original cued condition however would be equal. The detrended time course of the visual-target detection revealed a rhythmic profile with peaks at 4 and 8 Hz for invalidly cued detections. A 90° phase offset at 8 Hz was observed between the detection of the target at the cued location and the detection of a target on the same object, and an 180° offset at 4 Hz between the detection of the target at the cued location and on a different object. ***d***. Behavioral oscillations in the alpha band are nested within theta cycles (from Song et al., 2014). After central fixation, an irrelevant pre-cue (used to reset attention) and a varying stimulus onset asymmetry (SOA), a target (either a circle or a square) was presented with equal chance within either of two peripheral boxes. Participants were asked to respond to the type of target (square or circle). The behavioral response time course depending on the SOA followed a complex oscillatory pattern of alpha oscillations nested in theta cycles (alpha power fluctuates in a theta rhythm).

Even though direct evidence is lacking, some evidence of existing literature can be re-interpreted in the light of the proposed framework. Specifically, if a single alpha cycle determines that multiple parts of an object or multiple stimuli are combined into a single object percept, then a close temporal relationship—within one cycle and not across two cycles—would predict attribute integration (into a single object). For instance, it has been shown that when the onset of two simple stimuli, presented successively at a given position, occurs within a cycle of low frequency oscillations (mainly in the alpha band) they would be perceived as a single object (e.g., Samaha and Postle, 2015; Shen et al., 2019). Wutz et al. (2018) further showed that the instruction of the task could influence the frequency of oscillations in anticipation of the stimuli. If two stimuli are presented in a proximal temporal relationship and need to be integrated, then the frequency of anticipatory alpha oscillations would be slower than when the two stimuli need to be segregated to perform the task (see Fig. 2*a*; see also Sharp et al., 2022). The interpretation is that the two stimuli would have a higher likelihood of being processed within a single cycle if the frequency was slower due to the extended window of opportunity. In line with our framework, this points to information processed within one alpha cycle being considered as a single object (composed of multiple attributes). However, in a recent publication Buergers and Noppeney (2022) challenge, the view of low frequency-dependent stimulus binding. Using more than 1100 trials presented to 20 subjects, the authors tested whether pre-stimulus alpha fluctuations influenced the perception of one or two flashes combined with none, one or two sounds as single or multiple events. They found that the subject’s accuracy did not depend on the stimulus onset asynchrony in a way that would allow for the conclusion of an alpha fluctuation dependent stimulus binding. However, multiple authors have pointed out that the respective publication in fact does not challenge the idea of alpha fluctuation dependent stimulus binding, because (a) Buergers and Noppeney only investigated pre-stimulus alpha fluctuations, which by definition cannot allow conclusions regarding alpha fluctuations during stimulus processing as pre- and post-stimulus alpha fluctuations likely reflect two different processes (Kawashima et al., 2024); (b) opposing results using the very same methodology have already been reported (Noguchi, 2024) and (c) the reliability of the results provided by Buergers and Noppeney appears limited according to Venskus (2023). Indeed, as mentioned above, our framework suggests that task accuracy should be predicted by alpha phase. Future research needs to particularly focus on confirming this idea.

The general idea of our framework would predict that if indeed object attributes are integrated within a single alpha cycle, separate objects should be sampled in different alpha cycles (organized along the theta/delta cycle, see framework). In addition, in anticipation of the presence of a target object at multiple possible spatial locations, the attentional spotlight would sample the visual space at the alpha rhythm (note that this is different to visual search, where the target is not anticipated at certain locations). This kind of evidence for the role of alpha fluctuations in sampling objects or spatial position stems from the literature on attention both, at the behavioral and neural level. Behavioral studies, specifically designed to track the attentional sampling of the environment (e.g., by comparing detection rate for different target positions following a resetting cue), have demonstrated that such sampling indeed was rhythmic (Landau and Fries, 2012; Fiebelkorn et al., 2013) (but see Brookshire, 2021 for a critical view on this literature). For instance, at the behavioral level, Dugué et al. (2015) have shown that sampling each stimulus presented within the same visual quadrant, involving within hemisphere processes, occurred at ≈ 7–10 Hz. Senoussi et al. (2019) also demonstrated that two stimuli (i.e., objects) presented in the same visual quadrant of a computer screen were sampled successively at 10–11 Hz one after the other, i.e., in successive alpha cycles. Note, that behavioral rhythms can only be seen as a proxy for neuronal oscillations. Electrophysiology investigations have however also linked alpha and attentional sampling. Although Fiebelkorn et al. (2018) mostly emphasized the role of theta oscillations (≈ 4–5 Hz; see below), they reported a link between alpha oscillations and detection in the frontal eye field (FEF) as well as, in the lateral parietal cortex (LIP) during the theta phase, associated with impaired performance (see Fig. 3*C* of Fiebelkorn et al., 2018). Even more convincing evidence regarding the role of cortical alpha oscillations in rhythmic attention has been provided by Gaillard et al. (2020). The authors were able to precisely decode the position of the attentional spotlight across time from multi-unit activity (MUA) in the FEF, (see also Astrand et al., 2016). In a 100% valid cued spatial target detection task, they demonstrated that the attentional spotlight continuously samples the visual space at 8–12 Hz (Fig. 2*b*) and predicts behavior. This indicates that, although there was only one relevant location, the attentional spotlight was observed in other locations in different alpha cycles, which supports the idea of different alpha cycles possibly representing different object representations (here encoded in target locations). Note, this approach only worked for MUA and not using the power spectrum of the Local Field Potentials (LFPs). The MUA was locked to LFP alpha and also between the left and right hemispheres. However, the attention-related information was in anti-phase between the two hemispheres, which might have prevented the detection of a clear relationship between alpha oscillations using LFP and behavior in other studies that did not use a decoding approach (e.g., Fiebelkorn et al., 2018).

### Alpha cycles are linked to processing single-object attributes

The framework further predicts that the processing of attributes, occurring in the gamma band or through spiking activity, would be processed at different phases of a single alpha cycle. Again, direct evidence is lacking to a large extent (but see alpha–gamma phase coding in Bahramisharif et al., 2018), although a few papers at least indirectly addressed this question.

Most notably, the behavioral study by Fiebelkorn et al. (2013) found that within object sampling occurred at, 8 Hz with a 90° phase difference between the cued and same-object location (Fig. 2*c*). More specifically, subjects had to report the occurrence of a target that could either be located on the same visual object than the cue or on a different object, where the spatial separation for both conditions was kept constant. It could be shown that if the target was located on the same visual stimulus as the cue, detection performance fluctuated at an 8 Hz rhythm. This was in stark contrast to the between object sampling, which occurred at 4 Hz (see Section sec:sloẇfluc). Additionally, the behavioral study by Jia J et al. (2022) showed that the normalized reaction time could be mapped to a phase offset of an 8 Hz rhythm. Thereby, two distinct stimulus feature sets (high and low saliency) were expressed in a sort of mirrored pattern. Reaction times for high saliency stimulus features were shorter and earlier in the alpha phase as compared to low saliency features. This pattern was reversed for the late alpha phase, which indicates that each set of stimulus features was processed at a preferred alpha phase.

While the paper by Fiebelkorn et al. emphasized the within-object sampling component of the framework, the work by Jia J et al. adds the notion of a phase dependent (i.e., phase coded) preference of this feature processing.

### Slower fluctuations are involved in multi-object sampling/organization

The framework suggests that slower fluctuations, e.g., in the theta or delta frequency range, are involved in organizing the processing of multiple objects.

Similarly to alpha oscillations, there is no evidence directly addressing this claim. However, the literature on covert attention provided results that could be interpreted along those lines.

A high number of both behavioral and electrophysiological studies have recently revealed a link between theta oscillations and attentional sampling (Bosman et al., 2009; Landau and Fries, 2012; Fiebelkorn et al., 2013; Dugué et al., 2015; Huang et al., 2015; Jia J et al., 2017; Fiebelkorn et al., 2018; Harris et al., 2018; Kienitz et al., 2018; Spyropoulos et al., 2018; Mo et al., 2019; Re et al., 2019; Gaillard et al., 2020). In line with the current framework, behavioral evidence has been shown that the detection probability of spatially separated targets—mainly as they appear in different hemifields—resembled an ≈ 4 Hz rhythm mostly with a 90° to ≈180° phase difference (Fig. 2*c*; see also Song et al., 2014; Huang et al., 2015; Huang and Luo, 2020).

Interestingly, Senoussi et al. (2019) found in their behavioral study that objects presented in different visual quadrants were sampled at the theta rhythm following a reset of attention while, as mentioned above, the sampling of objects presented within a hemifield occurred at the alpha rhythm (see also behavioral results in Fiebelkorn et al., 2013).

The results of three behavioral experiments could be reinterpreted through phase coding to support the proposed framework. While further experiments are still needed, these reinterpretations offer a useful starting point. In a lateral attention task, Michel et al. (2022) found that guessing the position of the gap in a Landolt ring was modulated at the theta rhythm (peak at 4.8 Hz). Even though both, the valid and invalid (i.e., when target was on the uncued side) uncorrected data exposed a peak for theta and alpha fluctuations (9.6 Hz), the joint model (valid and invalid conditions combined) only reached significance for theta but not for alpha fluctuations, possibly due to cross-condition phase alignment for the former but not for the latter. In the light of the current framework, this would be interpreted as successful multi-object sampling within one theta cycle, which can be clearly distinguished from alpha based (within-object) sampling (see above). Here, the lack of a significant result for the joint model in alpha fluctuations would be interpreted as indirect evidence for an ≈180° phase offset, which would eliminate the effect in the joint model. Additionally, a very relevant result, supporting our framework, was reported by the group of Huan Luo. They presented stimuli with two saliency levels in different trials of the same blocks, at a similar position, and were able to show that the theta phase associated with better detection of the two stimuli was different (between 90° and 180°). Alpha phase also impacted detection performance, but no phase difference was observed between the two types of stimuli (Huang and Luo, 2020). Interestingly, such rhythmic and phase pattern was not observed when the two types of stimuli were presented in different blocks, indicating a role of rhythmic activity for organizing the sampling in the context of “competing” stimuli. In accordance with the framework, multiple objects (here multiple stimuli) appear organized in terms of saliency, such that the more salient stimuli were encoded earlier in the theta cycle as compared to the less salient stimuli (separated by a 90° offset). The absence of a phase offset effect in alpha fluctuations, indicates that - given the phase offset effect in theta - stimuli might have been processed in different alpha cycles, which would exactly follow the here proposed predictions. However, while Huang and Luo observed a theta effect for stimuli presented at the same location, Senoussi et al. did find alpha to be the dominant rhythm when two stimuli were presented in the same visual quadrant (not even at the very same location). In contrast to Huang and Luo, Senoussi et al. did not investigate the respective phase dependencies of both rhythms. A re-analysis of the data recorded by Senoussi et al. using a phase based approach (as used by Huang and Luo) might indeed yield comparable results. Furthermore, the main difference between both experiments is the definition of stimulus saliency. Huang and Luo manipulated saliency as part of their experimental setup, whereas Senoussi et al. determined “saliency” post-hoc as the probability with which each stimulus was reported correctly over the other (i.e., was more attended compared to the other) which again might explain the difference in the respective results.

The results presented in the two sections above suggest that one item is processed within one alpha cycle while multiple items are processed within one theta/delta cycle, but at different theta/delta phases. This indicates that slower fluctuations in the theta band are related to the organization of multi-position/ multi - object sampling, with each position/object being sampled in the alpha rhythm (Dugué and VanRullen, 2017; VanRullen, 2018; Fig. 1 bottom). This is also in line with the electrophysiological data of Gaillard et al. (2020) showing that, while behavioral performance followed both theta and alpha rhythms, the rhythm of shifts of the decoded attentional spotlight position in FEF was much stronger in the alpha rhythm.

Saccades themselves could also act as the driving mechanism for the slow changes of excitability during natural vision, because saccadic eye movement - although highly variable in time - has a mean frequency of around ≈3 to 5 Hz (Wutz et al., 2016; Leszczynski et al., 2021) and was shown to reset slow frequency fluctuations (Melloni et al., 2009). Furthermore, saccadic eye movement was shown to be related to the phase of alpha too (Pan et al., 2023; Shaverdi et al., 2023), which again would establish the link between slower fluctuations and alpha within the present context.

### Evidence for cross-frequency coupling between slow fluctuations, alpha and gamma oscillations

The framework introduced here implies, at the neuronal level, that synchronizations in the different frequency bands not only coexist next to each other and exchange information but are directly influenced by each other in the sense of nesting. This means that e.g., low frequency oscillations like theta and alpha fluctuations would jointly form the basis for visual scene processing.

First, we expect gamma oscillations to be nested within alpha oscillations. This has been demonstrated by several studies (Voytek, 2010; Park et al., 2011; Spaak et al., 2012; Bahramisharif et al., 2013; Bonnefond and Jensen, 2015; Popov et al., 2017; Bahramisharif et al., 2018; Pascucci et al., 2018; Seymour RA et al., 2019; Yang et al., 2024).

The framework further predicts a coupling between theta frequencies and alpha frequencies (see also Dugué and VanRullen, 2017). This specific coupling has generally been rarely investigated due to difficulties in signal processing as alpha frequency is within the harmonic range of theta frequency.

Song et al. (2014) for instance have revealed that the behavioral detection of a target was following a pattern approximating a coupling between alpha and theta oscillations (Fig. 1 bottom). Also at the behavioral level, Fiebelkorn et al. (2013) revealed a coupling between the 4 Hz and the 8 Hz rhythm at the same object location (figure S2 of Fiebelkorn et al., 2013). The EEG study by Jia J et al. (2017) revealed that the temporal response function, i.e., perceptual echoes, associated with the two stimuli presented in different hemifields was expressed in the alpha-band, as previously shown (VanRullen and Macdonald, 2012), but was switching between the two displayed stimuli at ≈ 4 Hz. Interestingly, Bellet et al. (2017) showed that detection performance (reaction times) in humans (1) follows microsaccadic timing, which exhibits a theta rhythm and (2) are related to the alpha/beta band’s phase(s). Additionally, these oscillations were pulsed sequentially across visual hemifields relative to the microsaccade direction, at a slow rhythm, first occurring in the same hemifield as the eyes move and then in the opposite hemifield. Finally, a direct coupling between theta phase and alpha power in LIP but also between theta phase in FEF and alpha power in FEF (at the unattended location) and LIP (at the attended location; see also the coupling with lower frequency at the unattended location) has been reported (Fiebelkorn et al., 2018). More generally, a recent study revealed that alpha fluctuations could be coupled to theta frequency in many brain areas in humans (Halgren et al., 2018). In addition, as predicted by the framework, a coupling between delta and alpha fluctuations has further been observed (see e.g., Gomez-Ramirez et al., 2011; Wilson and Foxe, 2020).

Further work needs to be conducted to specifically test the multi-level frequency nesting, where gamma is predicted to be nested in alpha fluctuations which in turn would be nested in slower frequencies like theta.

### Frequency adaptation

The nature of the proposed framework implicitly assumes that there is a limit of information to be processed within a low-frequency oscillation cycle (since e.g., each attribute belonging to a single object must fit into one cycle). This further implies that in order to increase or decrease the information content (per cycle), the respective cycle length needs to be adjusted. In general, this would be possible in multiple ways: the most straightforward way of fitting more information into a single cycle would be to lower the respective frequency, which directly widens the window of opportunity for any bit of information to fall into the respective cycle. Another more indirect way would be to keep the frequency constant but change the amplitude, i.e., the duty cycle. Considering e.g., the inhibitory nature of alpha fluctuations, a way to narrow down the window of opportunity would be to increase power which would steepen the inhibitory gradient per time unit (effectively shortening the time of low inhibition).

Directly complying with the predictions of our framework, Ronconi et al. (2017) found that the complexity of stimuli to be integrated is inversely related to the exact frequency of slow oscillations related to performances. In addition, the difficulty of the task or the number of stimuli have been shown to influence the frequency involved (e.g., behavioral results by Holcombe and Chen, 2013; Chen et al., 2017). The more complex a stimulus, the lower the frequency of the underlying rhythm. As mentioned above, Wutz et al. (2018) further showed that the task instruction could influence the neuronal frequency of oscillations in anticipation of the stimuli. Specifically, in anticipation of the stimulus, the cycle length was shortened (by increased frequency) if the task required stimulus segregation and lengthened (by decreasing frequency) if the task required stimulus integration (Fig. 2*a*; see also Sharp et al., 2022).

On top of that, a very common indirect finding is the strong variation in exact alpha and theta peak frequency reported in the literature, which potentially could be explained by varying stimulus complexity and/or experimental instructions in those different studies.

More importantly, a recent paper has raised the issue of the analyses performed on behavioral data, which might artificially generate peaks in the power spectrum while the dynamic of attention would be aperiodic (Brookshire, 2021). As previously mentioned, our model does not state the necessity of rhythmical activity per se (although, neither does our model exclude this possibility), but rather highlights the dynamic processes involved in this hierarchical phase coding scheme.

The neuronal mechanism supporting complex visual processing must therefore be studied, considering highly dynamic and even burst-like events in order to shed light on the underlying computational principles accurately (see Jones, 2016; Eliav et al., 2018; Bush and Burgess, 2019).

### The influence of the “zoom lens of attention”

So far, the terms “object” and “attribute” have been merely used as implicitly defined entities. However, as mentioned in the introduction, the definition of an object might vary depending on what is within the focus of attention. Considering the scene in Figure 1, the focus of attention could cover the entire scene and the cat would be considered an object with its attributes head or paws. However, if the focus of attention changes, then the head of the cat and its ears or eyes become objects and the shape of its eyes or ears the new attributes. Eriksen (1986) described this idea as the “zoom lens of attention,” where the size of the attentional spotlight determines the level of detail at which respective parts of an image are processed. Within the presented framework, the level of detail that can be processed is limited by the cycle length of either delta/theta (number of objects) or alpha fluctuations (number of attributes per object). A wide attentional spotlight comprising more objects would lead to a lower theta frequency and possibly power (to be able to sample more objects within a single cycle), but higher alpha frequency to fit more alpha cycles in one theta cycle. This would mean that the level of detail is reduced, because less attribute information could be sampled. On the contrary, for a narrow attentional spotlight (the zoomed in state), theta might expose a higher frequency in order to sample the respective objects quicker (or more often, depending on the task), whereas alpha fluctuations might decrease in frequency and power in order to fit more attributes into a single cycle. Both would increase the level of detail processed by accumulating more information. Note that this would further interact with other external demands such as task instructions (see above) which could limit the amount of time each object could e.g., be viewed.

While conclusive evidence for a direct link between the quality of information and the respective “zoom factor” is lacking, some indirect evidence suggesting an increased level of detail at higher zoom levels (smaller fraction of the scene is viewed) and vice versa is discussed below.

A first publication serves as indirect evidence for the idea that indeed a narrow attentional spotlight leads to narrow but strong cortical activity and a wide attentional spotlight to lower but more spatially distributed cortical activity. Müller et al. (2003) found that the more items were cued as potential target locations on the screen (as a measure for the width of the attention spotlight or the zoom), the lower the observed BOLD response in primary visual regions (V1, V2, VP and V4) indicating a more coarse stimulus processing. In turn, the peak BOLD response was inversely related to the size of the attentional spotlight, such that the highest response in each region was observed for the smallest number of cued target locations. Again, this indicates that information was processed in more depth but at the expense of a smaller spatial extent. The presented results reflect the more quantitative perspective of the relationship between neuronal activity and the size of the attentional spotlight. For the presented framework, we would expect alpha band cortical dynamics to act similarly. If indeed a small attentional spotlight reflects increased processing of object attributes, then alpha band fluctuations should be spatially narrow and limited to the cortical areas processing the respective part of the scene that is in focus. Furthermore, the opposite should be observed for wider attentional spotlights. Feldmann-Wüstefeld and Awh (2020) attempted such an experiment using surrogate EEG channels and indeed report that the spatial extent of alpha sources is positively related to the size of the attentional spotlight, which is in turn negatively related to the strength of the alpha effect across the different channels. This publication however does not address the level of detail but only the width of the attentional spotlight.

In addition, we expect an interaction between stimulus resolution and attention spotlight. Itthipuripat et al. (2014) found that while a narrow attentional spotlight enhanced the neural response gain (i.e., preference) to high contrast stimuli, a wide attentional spotlight enhanced the neural response gain to middle-contrast stimuli. Hence, for small attentional spotlights, neuronal populations tend to prefer high contrast stimuli (indicating an increased level of detail or resolution), whereas for wide attentional spotlights, neuronal populations tend to prefer lower contrast stimuli.

We further assume that the relationship between size of attentional spotlight and alpha band activity similarly extends to the processed stimulus resolution, with increased resolution for smaller attentional spotlights and vice versa. In order to fit more information into a single cycle, one possibility—as discussed above—is to reduce the underlying frequency. In turn, for a more local, detailed processing a faster frequency would support the separation of small details by distributing objects or object attributes across cycles. Furthermore, a more global processing would hence involve the processing of distributed objects (accompanied by a change in theta/delta), whereas a more local processing would rather favor the processing of object attributes (accompanied by a change in alpha fluctuations). Indirect evidence from Smith et al. (2006) points towards this direction. For ambiguous images (like Dali’s “The Slave Market with Disappearing Bust of Voltaire”) Smith et al. (2006) report a relationship between a more global processing of the image (Voltaire) and slow frequency theta fluctuations and a more local processing (the nuns) that was linked to beta band fluctuations (see also Romei et al., 2011).

So far the presented literature provides partial evidence that slow theta/delta and alpha fluctuations (potentially even beta) band activity are indeed linked to the size of the attentional spotlight and the level of detail at which the respective visual input is processed given the respective size. Most crucially, however, it remains to be understood how the change of reference frames actually affects the underlying frequency architecture. It is possible that when a change of reference frame from multiple to single object processing that requires an increased resolution is required, the former theta/delta related multi-object sampling, now applies to the single object that is required to be processed at higher resolution, since what has formerly been referenced as attributes per object now becomes the new object collection with more detailed new attributes that in turn would be linked to alpha band activity (see the comparison between Fig. 1 left & right). Hence, the spatial extent at which theta/delta is expressed would be shrunk to the extent that was linked to alpha fluctuations before when “zooming in”. Alpha oscillations in turn would potentially be expressed at an even smaller spatial scale, since the level of detail has to be increased at the cost of spatial extent.

## Role of prior knowledge and active inference

For the proposed framework to function as described, it is required that prior knowledge of objects and attributes that typically belong to certain objects is available. Hence, this framework could be seen as somewhat related to predictive coding framework. Note however that the absence of prior knowledge does not necessarily break the framework, since prototypical low level attributes (e.g., simple geometrical shapes like lines) are potentially “hard-coded” in the neocortical structure (e.g., oriented bars in V1).

In a nutshell, the predictive processing scheme suggests that perception reflects an inferential process requiring the interactions between prior/perceptual hypotheses flowing in the feedback direction, and sensory evidence, flowing in the feedforward direction (Harkness and Keshava, 2017). The deviation between prior and input is termed prediction error and generates a neuronal response pattern which allows updating the prior (Friston, 2019; Parr and Friston, 2019). Clear hypotheses about the computational role of specific oscillations, expressed in specific layers of the cortex, have been and can be further derived from the literature (Sedley et al., 2016; Chao et al., 2018) and recent frameworks (Bastos et al., 2012; Fries, 2015; Bonnefond et al., 2017; Xiong et al., 2023). For instance, predictions and prediction errors would be encoded in beta and gamma oscillations respectively (e.g., Bauer et al., 2014; Sedley et al., 2016; van Pelt et al., 2016; Bastos et al., 2020). Interestingly, Fiebelkorn et al. (2018)’s results could be reinterpreted in the light of this predictive coding scheme. In anticipation of a target, they observed beta activity, during the “good” theta phase in FEF, sent downwards to LIP possibly reflecting the prediction about the anticipated target while gamma was also observed during this phase in LIP and sent to FEF possibly reflecting the prediction error related to the absence of target during this anticipatory period.

How do the results and the framework presented above relate to these ideas? In a first step, a prediction about the upcoming visual scene would already tune the respective theta/delta and alpha activity towards a preferential processing. For the example above, a coarse expectation (or prediction) would be formed when leaving the house and entering the garden, where you expect to see your cat. Since the overall structure of the garden and the cat’s favorite spot are known, this coarse prediction already allows for a preparatory setup of the slow frequency machinery.

In the second step, the first feed-forward sweep of sensory information provides evidence of the actual visual perception. We suggest that predictions about (anticipation of) the objects to be processed are formed during the late phase of the theta/delta fluctuation (but see Jensen et al., 2021) and then routed down the hierarchy in the form of beta bursts during the post-inhibition (or post-saccade) phase. This information is then compared with the actual sensory input and produces prediction-error related gamma band activity for each of the predicted attributes within an alpha cycle. Thereby, a violation of an attribute prediction could be considered a partial violation of the object prediction as well. This means that a prediction error caused by one attribute (linked to alpha oscillations) would inevitably cause a prediction error related to the theta/delta cycle as well. However, if the object prediction is violated by e.g., changing the object, the respective prediction error would mostly be linked to theta/delta and only coarsely to alpha oscillations since the predicted attributes could not be compared against evidence.

Similar to gamma, and in line with the idea developed here, alpha–beta coupling has also been reported (Grabot et al., 2019). However, a more formalized framework including the relationship between cross-frequency coupling in the light of predictive coding that also includes object and attribute sampling remains to be developed (see also Alamia and VanRullen, 2019). In general, a prediction derived from the framework would be that alpha and theta phase depend on priors. We suggest that having accurate priors about the stimulus properties should modulate alpha/theta phase accordingly and facilitate the processing of the stimulus and its elements, and therefore be associated with better performance. Integration/segregation (Wutz et al., 2018) need to be linked to the respective phase given certain priors (e.g., via task instructions).

## Relation to previous phase coding frameworks

The ideas presented here pertain to other phase coding frameworks developed for spatial cognition (O’Keefe and Recce, 1993), working memory (Lisman and Jensen, 2013) or the prioritization of visual stimuli (Jensen et al., 2012, 2014; Bonnefond et al., 2017). Phase coding has been intensively studied in place (and grid) cells, that is, hippocampal and entorhinal neurons that fire as a rat runs through specific locations in space. Specifically, it has been shown that these cells fire at specific phases of the ≈ 8 Hz theta oscillations present during locomotion in rats, and that the firing precesses to earlier phases as the place fields are traversed. Note that it has been shown that rodent hippocampal theta centres around 8 Hz while human hippocampal theta during spatial navigation is slower at ≈ 3–4 Hz (Watrous et al., 2013; Jacobs, 2014). In addition, multiple functionally different low- and high-frequency theta rhythms have been identified to serve different purposes even within the hippocampus (Goyal et al., 2020). It has also been shown that the maintenance of working memory representations of different objects, reflected by high-frequency gamma activity, would occur at distinct phases of theta or alpha oscillations (Heusser et al., 2016; Bahramisharif et al., 2018). What we suggest here relates to these ideas, by suggesting that the different elements of a single object are processed at different phases within one alpha cycle, the most salient (due to specific features such as a higher contrast) or attended attributes (through covert or overt attention) being processed first as it would be able to earlier overcome the pulsed inhibition (Fig. 1). While, Jensen et al. (2014) suggest that different objects would be observed at different phases of an alpha cycle, we rather predict that different attributes of the same object observed within alpha oscillations. The processing of the different objects would occur at different phases of the slower fluctuations.

## Avenues for experimental validation

This framework can be tested using multiple approaches, such as MEG in humans, to obtain an overview about dynamic brain networks. Additionally, laminar electrode recordings such as LFPs / MUA, separating excitatory and inhibitory neurons in multiple visual regions along the visual hierarchy, might provide a more detailed insight. To test the hypothesis that the different elements of one object are processed within an alpha cycle, the design should allow to separate activation of two elements (possibly over hemisphere for MEG recordings). Manipulating the relevance or saliency of the different elements would allow determining whether processing, as indexed by gamma or MUA, would occur at different phases according to relevance or saliency. To test the role of slower frequencies, the number of objects presented simultaneously, as well as the relevance or saliency and the possibility of performing saccades or not could be manipulated. This would allow us to determine the role of slower rhythms in organizing the processing of each object. We predict a decrease in frequency with increasing complexity and that the order of objects processed along the phase (i.e., phase coding) depends on object’s relevance or saliency. Montemurro et al. (2008) found in monkeys that during naturalistic stimulation (color movies) 54% more information was obtained by considering the LFP phase at which spikes occurred as compared to spike count alone. We would predict that this additional information could be separated in within and between object information depending on whether the underlying LFP frequency would be in the alpha or theta range respectively.

Another way to test the framework from existing paradigms would be to modify designs presented in Figure 2*b,c* (Fiebelkorn et al., 2013; Gaillard et al., 2020). For instance, if two bars were presented instead of four squares in Gaillard’s design (Fig. 2*b*), we would expect the different parts of the bars to be decoded at different phases of a single alpha cycle and not in different alpha cycles. On the contrary, if four squares were presented the paper by Fiebelkorn’s paper (Fig. 2*c*), we would predict that the target detection rate would only fluctuate in a theta rhythm with a different phase for the different target positions.

Furthermore, the non-sinusoidal nature of the signal have to be considered. Methods such as cycle-by-cycle analysis (Cole S and Voytek, 2019) or empirical mode decomposition (Liang et al., 2005) can thus be used to overcome the assumptions of stationarity and sinusoidal shape that come with traditional Fourier transforms. This would allow researchers to rule out more precisely at which exact moment the underlying inhibition could be overcome, but would not affect the framework’s prediction that certain types of information (e.g., stimulus features) would be grouped along the same gradient.

## Open questions

At a general level, this framework heavily relies on the idea of phase coding at different frequencies (e.g., theta/delta and alpha oscillations) which remains to be fully addressed (but see O’Keefe and Recce, 1993; Bahramisharif et al., 2018).

One of the predictions produced by our framework is that alpha oscillations allow the grouping of the processing of different attributes of one object (Fig. 1 left). A related prediction would then be that alpha oscillations in e.g., deep layers of cortical columns involved in the processing of each element of an object should be synchronized (see Mima et al., 2001 for evidence in line with that prediction). In addition, multiple objects should be linked to alpha oscillations in different cycles, whereas multiple attributes of a single object should be linked to alpha oscillations within the same cycle.

Our model predicts that the frequencies involved in processing the attributes of an object (in the gamma-range), the object itself (in the alpha-range) and the organization of the processing of multiple items (in slower frequency range, including saccadic timing) might dynamically vary depending on the features of the elements/objects or cognitive demand for flexible and adapted processing of complex visual scenes (Fig. 1). Manipulating complexity should therefore be associated with a change in the peak frequency or the spread of frequencies, and potentially influences the onset timings (and frequency) of saccades. However, the analyses commonly performed in our field are possibly not adapted to test the predictions of the framework as complex and dynamic scenes would be associated with multiple frequency changes preventing the clear detection of a prominent peak in the power spectrum using e.g., over trial averages (see Eliav et al., 2018; Bush and Burgess, 2020). The literature on the detection of beta bursts or waveform shape might provide a basis for developing adapted tools (Cole SR and Voytek, 2017; Shin et al., 2017).

Furthermore, the relationship between saccadic eye movement and foveal and parafoveal processing needs to be further investigated. One possibility would be to test whether stimuli presented at the fovea and parafovea are preferentially processed within the same alpha cycle (Jensen et al., 2021) or in a different alpha cycle relative to the onset of the saccade.

As scenes are dynamic, the motion of objects should be further integrated to perceive the scene as coherent. Only binding between motion and color has been studied and has been related to alpha oscillations in monkeys (Dezfouli et al., 2021) and EEG/ tACS studies (Zhang et al., 2019; Ghiani et al., 2021). We predict that alpha oscillations would further be involved in integrating motion and perceiving one moving object as remaining the same object via synchronization between regions specifically processing the identity of the objects and MT region which is specifically associated with processing motion.

This framework has to be further related to brain communication frameworks. It has been proposed that alpha coherence would implement the routing of information reflected in the gamma oscillations (see Bonnefond et al., 2017). Here, between areas alpha synchrony could allow the information related to the different attributes to be routed in the hierarchy. The role of the pulvinar which is connected to numerous areas in the visual hierarchy is expected to be crucial in controlling this alpha oscillations related flow of information (Saalmann et al., 2012; Fiebelkorn et al., 2019) as well as the claustrum (Wang Q et al., 2017). The question remains whether we would expect theta oscillations to play a role in network communication. Theta oscillations would originate in the FEF/LIP network and influence activity in visual areas directly or through the dorsal and/or ventral pulvinar (Arcaro et al., 2018; Saalmann et al., 2018). FEF/LIP could therefore exert a theta-rhythmic modulation of activity in the early visual cortex, which would then be propagated through the hierarchy of the visual network (but see Kienitz et al., 2018; Fiebelkorn et al., 2019). Further investigations of the interaction between the fronto-parietal network, the visual network, the claustrum and the pulvinar are necessary.

Those interactions could furthermore be implemented in the form of traveling waves. In that sense, the information would be transmitted as part of the moving phase gradient implemented in the traveling waves and would lead to a similar separability of objects and features in the target region as has been proposed for spatial information (Lozano-Soldevilla and VanRullen, 2019). Furthermore, it could be shown that the directionality of traveling waves depended on whether episodic memory was encoded or retrieved (Mohan et al., 2024). This gives rise to the interpretation that traveling waves in the current framework could reflect the interface between sensory information and knowledge in terms of predictions (Alamia and VanRullen, 2019; but see Zhigalov and Jensen, 2023 for a discussion regarding the nature of these traveling waves).

Are similar mechanisms involved in other modalities? Language processing involves the binding of information over time (across syllables to form words, across words to form sentences) that could be processed within cycles of e.g., delta or theta-like fluctuations (Ding et al., 2016). Although languages exhibit statistical regularity that we learn through development, the dynamic of any language is not perfectly rhythmical. Therefore, it might also require very dynamic adjustment of slow fluctuations involved in processing of single syllables/words and sentences. Finally, other rhythmical motor-related activities such as sniffing or breathing might also influence the rhythmical processing of stimuli and control faster oscillations/fluctuations (Schroeder et al., 2010; Wachowiak , 2011; Klimesch, 2018; Perl et al., 2019).

## Concluding remarks

A central question in (cognitive) Neurosciences is indeed whether brain oscillations (we preferred to use the term “fluctuations” to better embrace their dynamic nature) are epiphenomenal or whether they are essential for orchestrating computations in the brain. It is therefore crucial to identify such computational principles like the ones described in this paper in order to test the active involvement of neuronal “oscillations” for visual perception.

## Synthesis

Reviewing Editor: Arianna Maffei, Stony Brook University Decisions are customarily a result of the Reviewing Editor and the peer reviewers coming together and discussing their recommendations until a consensus is reached. When revisions are invited, a fact-based synthesis statement explaining their decision and outlining what is needed to prepare a revision will be listed below. The following reviewer(s) agreed to reveal their identity: Rober Boshra.

The reviewers agree that the manuscript provides an interesting and novel perspective on the relationship between neural oscillations and visual scene processing. A few inconsistencies in the presentation of the framework were identified and some descriptions of the ideas was found to lack clarity.

The specific comments of each reviewer are reported below.

Reviewer #1

I did notice one point that the authors may want to address. Specifically, they first discuss a paper in which a null results suggests, "They found that the subject's accuracy did not depend on the stimulus onset asynchrony in a way that would allow for the conclusion of an alpha fluctuations dependent stimulus binding."

However, in the section on predictive coding, they state, "In a first step a prediction about the upcoming visual scene would already tune the respective theta/delta and alpha activity towards a preferential processing."

Perhaps these two ideas should be integrated since having an accurate prior would likely lead to faster performance.

Also, the authors could perhaps discuss specific neural substrates in more detail. Data from several structures is mentioned. But it seems unlikely that hierarchical scene processing would be a property of early visual cortex. Or for that matter, parietal cortex. Perhaps the parahippocampal place area? Maybe a paragraph discussing specific neural substrates for the claims would be helpful. Particularly to guide future research.

Related to this, it would be helpful to lay out specific experimental predictions at the end. What experiment could be done, and which specific measurements made, to support/refute the hypothesis. Would LFPs or MUAs be the best way to test these ideas? What behavior would be best?

Reviewer #2

The authors propose an intriguing framework that synthesizes prior visual processing and attention literature. The authors show how nested multiplexing of representations in the theta, alpha, and gamma bands may enable the brain to process complex multi-object scenes. The general premise of the proposed framework is appealing and the synthesis of what may seem like incompatible results is quite valuable; however, I believe that further work needs to be done to refine the manuscript for a clearer, well-constrained write-up of the authors' ideas. I list my comments below:

-The phase-dependent inhibition aspect of alpha rhythms is not intuitive without reading the Jensen et al reference (e.g. "Strong signals are able to overcome the pulsed inhibition earlier in the cycle than less strong inputs"). The authors should expand on this in the manuscript for readers as it is an important consideration for the framework. A visual that integrates this inhibition within the nested cycles of alpha/theta/gamma may be useful.

-In reading the paper, I struggled with disentangling between behavioral and neural oscillations. While I think it's reasonable to link behavioral rhythms as a manifestation of underlying neural rhythms, care should be taken as to where, anatomically, these neural rhythms have been reported from, especially to support alpha-theta rhythms. For example, if you examine spectral power across the human brain in electrocorticography, it is common to find a gradient of slowing spectral peaks that is more alpha-like in posterior/occipital areas that slows to theta-like oscillations as it gets to prefrontal areas (e.g. https://doi.org/10.1016/j.neuron.2018.05.019). These oscillations take place during the same tasks and may directly interact with multi-object representations. Integrating these varying alpha/theta/gamma oscillations across areas within the authors' framework will be greatly useful to the reader.

-Stemming from the earlier point, could the authors speculate on how traveling waves may interact with their framework?

-In the framework, a single object is represented as one alpha cycle. Have the authors considered what the implications would be (as observed in behavioral and neural oscillations) if the two tasks in Fig. 2B and 2C are converted to have the same parameters (e.g. 75% valid trials, endogenous cueing) with the only difference being that the task in B would have 2 objects (bars) and the task in C would have 4 (squares)? I believe that this would be a simple manipulation that may directly support the proposed framework.

-The authors detail several aspects of the Michel et al., 2020 study. My understanding is that this is a conference abstract so it was difficult to follow the exact findings before contextualizing within the proposed framework. Further detail would help or a more detailed reference.

-The authors brought up an excellent point in the abstract regarding the possible non-sinusoidal nature of these rhythms and possible modulations on wave-shape. It was not intuitive to me how this integrates within their framework later in the manuscript. An elaboration of that in the implications will be very relevant, especially that if particular phases are stretched or if the sinusoid is altered, it is not difficult to imagine the central frequencies of these rhythms to change when examined by the fourier transform. Does the authors' framework predict that a non-sinusoidal basis for analysis would generate different results? (e.g. empirical mode decomposition or algorithms proposed by the Cole &Voytek reference)

-The authors define alpha to be within 7-14Hz, but they later discuss 8Hz as a theta cycle. A clear distinction should be made to the reader if one exists. If not, a brief discussion on the spectral "boundaries" between these two low-frequency bands would be valuable.

-In the caption of Fig. 2, the first panel is listed as from the 2014 Wutz paper. In checking the reference, I believe this may have been mixed with the 2018 PNAS study.

-Figure 2 and its caption will benefit from refinement for clarity. Explicit identification of each component in each panel may help. Also, what is the time-locking event in the time-frequency spectrum of panel D?

-The paper will benefit from some punctuation/typo edits (e.g. see missing section in line 279)
